# Non-invasive methods to assess muscle function in dogs: A scoping review

**DOI:** 10.3389/fvets.2023.1116854

**Published:** 2023-01-30

**Authors:** Kathrine Højte Dahl, Mette Kreutzfeldt Zebis, Anne Désiré Vitger, James Edward Miles, Tine Alkjær

**Affiliations:** ^1^Department of Veterinary Clinical Sciences, University of Copenhagen, Copenhagen, Denmark; ^2^Department of Midwifery, Physiotherapy, Occupational Therapy and Psychomotor Therapy, University College Copenhagen, Copenhagen, Denmark; ^3^Institute of Sports Medicine Copenhagen, Copenhagen University Hospital–Bispebjerg and Frederiksberg, Copenhagen, Denmark; ^4^Department of Biomedical Sciences, University of Copenhagen, Copenhagen, Denmark; ^5^The Parker Institute, Bispebjerg-Frederiksberg Hospital, Copenhagen, Denmark

**Keywords:** dogs, muscle function, methods, non-invasive, cranial cruciate ligament rupture

## Abstract

Skeletal muscle function can be affected by multiple disorders in dogs of which cranial cruciate ligament rupture or disease (CCLD) is one of the most common. Despite the significance of this condition only sparse research exists regarding assessment of muscle function in dogs. This scoping review aimed to identify the non-invasive methods for canine muscle function assessments that have been reported in the literature in the past 10 years. A systematic literature search was conducted 1^st^ March 2022 across six databases. After screening, 139 studies were considered eligible for inclusion. Among the included studies, 18 different muscle function assessment categories were identified, and the most frequently reported disease state was CCLD. We included an attempt to elucidate the clinical applicability of the 18 reported methods, as experts were asked to subjectively assess the methods for their clinical relevance as well as their practical applicability in dogs with CCLD.

## 1. Introduction

### 1.1. Rationale

Impaired skeletal muscle function is frequently a consequence of orthopaedic disorders. A positive outcome for orthopaedic disorders is dependent on optimal return to normal muscle function. One of the most common orthopaedic disorders causing lameness in dogs is cranial cruciate ligament disease (CCLD) (1–5). In CCLD, the function of the skeletal muscles is particularly challenged, as the normal stabilising synergism between the large muscles around the canine stifle joint and the cruciate ligaments is disrupted (1). It has been found that the extension and flexion angles of surgically treated CCLD stifles are inferior to healthy joints, and that the active range of motion is impaired in the surgically treated limb at long-term assessment (6). Further, it has been found that 6–18% of dogs seen by a veterinarian for reasons related to CCLD are subsequently euthanized due to this diagnosis (3, 7). Reasons for euthanasia include treatment costs and the risk of persistent lameness (3, 6, 7). Due to the incidence and long-term consequences of CCLD, it is critical for surgeons and physiotherapists to have tools to evaluate the functional capacity of patients. Muscle function evaluations could help target therapy and thereby improve the outcome of surgical interventions.

Skeletal muscle function can be measured and evaluated in different ways. Muscle activity can be evaluated by e.g., electromyography and acoustic myography (AMG), the latter also called mechanomyography (8). While muscle mass and tone may be estimated by simple palpation, objective information about mass and muscular health can be obtained from multi-frequency bio-impedance or ultrasound (9, 10). Magnetic resonance imaging (MRI) and computed tomography (CT) are other imaging methods available to evaluate muscles (11, 12). However, a major disadvantage of these two methods is the necessity for the animals to be anaesthetized or at least heavily sedated to ensure that they do not move (13, 14). Muscle function can also be evaluated biomechanically e.g., gait analysis where estimations of kinetic–and kinematic parameters quantify the patients' ambulation (15, 16).

The role of skeletal muscles in orthopaedic diseases such as CCLD emphasises the clinical relevance to muscle function assessment in dogs and an overview of methods for this purpose would be helpful. A scoping review is an approach to systematically identify and map the literature on a specific topic in a replicable way (17, 18). No previous scoping reviews of muscle function assessment in dogs exist. Thus, it is appropriate to use this approach to identify and categorise the available literature on this topic.

### 1.2. Objectives

The objective of this scoping review was to identify and map the primary research literature on non-invasive methods for assessing muscle function in dogs using formal scoping review methodology. In addition, we also aimed to evaluate the relevance and applicability of the methods in clinical settings, in relation both to their value as muscle function assessors and to the complexity and costs of these procedures.

## 2. Methods

The scoping review followed the Guidance for conducting systematic scoping reviews from Joanna Briggs Institute (19) and was reported according to the PRISMA Extension for Scoping Reviews (PRISMA-ScR) with subheadings corresponding to the recommendations (17).

### 2.1. Protocol and registration

Our protocol for the current scoping review followed the Preferred Reporting Items for Systematic Reviews and Meta-Analysis: Extension for Scoping Review Guidelines (PRISMA-P) 2015 statement (20). The final version of the protocol was uploaded and registered prospectively in the Open Science Framework 1^st^ March 2022 (https://osf.io/r7ja2).

### 2.2. Eligibility criteria

For studies to be included in the scoping review, the study population had to be live dogs or other animals in the canid family (foxes, wolves etc.). Studies had to address a muscle function assessment method. The context element of this scoping review was limited to non-invasive clinically relevant settings. Thus, studies were excluded if the muscle function assessment involved an invasive step (e.g., needle electrodes, blood samples, sedation, or anaesthesia). Studies comprising both invasive and non-invasive settings were included if these were adequately described to make it clear which parts of the study were eligible.

Language was restricted to English, and publication date to the last 10 years (2012-) to ensure current applicability in clinical settings. Book chapters and reviews were excluded to avoid subjective opinions and the risk of double reporting. When we identified reviews, we checked the reference list for eligible studies and included them if they were missed by our search.

### 2.3. Information sources

To identify potentially relevant studies, a systematic literature search was conducted 1 March 2022 across the following bibliographic databases: Web of Science (RRID:SCR_022706), CAB Abstracts (CABI) (RRID:SCR_016467), Ovid MEDLINE® ALL (RRID:SCR_002185), AGRICOLA (RRID:SCR_008158), Scopus (RRID:SCR_022559) and Embase (RRID:SCR_001650). The databases were searched from 2012 to 1^st^ March 2022. The search strategy was discussed and refined through team discussions and in collaboration with experienced librarians from Copenhagen University Library. The search results for all six databases were exported to the reference manager software EndNote (EndNote™ version 20, Clarivate, Philadelphia, USA, RRID:SCR_014001), and duplicates were removed. When online access to studies was not possible, full texts were requested by contacting the author(s) *via* either ResearchGate [www.researchgate.net, an academic social network, (RRID:SCR_006505)] or by personal inquiry *via* e-mail.

### 2.4. Search

The literature search was prepared using preliminary searches of Web of Science and Ovid MEDLINE® ALL to identify studies on the topic. The relevant records were then analysed for specific words contained in the title or the abstract, and these words were used to develop the full search strategy. The final literature search performed (by KHD) 1^st^ March 2022 is shown in Table 1 and examples of the full electronic search strategy (for Embase and Web of Science) can be found online in Appendix I (https://osf.io/sdnxj).

**Table 1:** Search strategy for the databases: Web of Science, CAB Abstracts, Ovid MEDLINE® ALL, AGRICOLA, Scopus and Embase.

			**AND**
OR	Dog^*^	Assess^*^	(Musc^*^ OR neuromusc^*^) funct^*^
	Canin^*^	Measure^*^	(Musc^*^ OR neuromusc^*^) contract^*^
	Hound^*^	Technique^*^	(Musc^*^ OR neuromusc^*^) recruit^*^
	Canis	Screening^*^	(Musc^*^ OR neuromusc^*^) work^*^
		Diagnostic^*^	(Musc^*^ OR neuromusc^*^) perform^*^
		Record^*^	(Musc^*^ OR neuromusc^*^) activ^*^
		Evaluat^*^	(Musc^*^ OR neuromusc^*^) characteri^*^
		Understand^*^	(Musc^*^ OR neuromusc^*^) condition^*^
		Analy^*^	(Musc^*^ OR neuromusc^*^) propert^*^
		Investigat^*^	(Musc^*^ OR neuromusc^*^) alter^*^
		Determin^*^	(Musc^*^ OR neuromusc^*^) destruct^*^
		Examin^*^	Myopath^*^
		Diagnos^*^	(Musc^*^ OR neuromusc^*^) deficien^*^
		Descri^*^	(Musc^*^ OR neuromusc^*^) disease^*^
			(Musc^*^ OR neuromusc^*^) force^*^
			(Musc^*^ OR neuromusc^*^) strength^*^
			(Musc^*^ OR neuromusc^*^) disorder^*^

Limitations were English as the language and year of publication from 2012 to 2022.

### 2.5. Selection of sources of evidence

All records retrieved were exported from EndNote and imported into Covidence systematic review software, Veritas Health Innovation, Melbourne, Australia (available at www.covidence.org, RRID:SCR_016484). Additional duplicates were automatically removed by Covidence before starting the screening process.

Initially, the reviewers aligned their understanding of the inclusion and exclusion criteria to increase consistency of the review process. Two independent reviewers (KHD and TA) reviewed the titles and abstracts of all the records for eligibility. A third independent reviewer (MKZ) evaluated discrepancies. Subsequently, the three independent reviewers (KHD, TA and MKZ) went through the potentially eligible records, and decided the final included records based on a full-text analysis. Each of the reviewers screened 2/3 of the total number and shared half of their screening with each of the two other reviewers. The whole screening process in Covidence was blinded, also for the reviewers themselves when discrepancies had to be solved. Reasons for exclusion were determined for each study: Invasiveness due to anaesthesia/sedation, invasiveness due to other conditions than anaesthesia (e.g., blood samples or electrodes), wrong outcome or intervention, inadequate description of intervention (e.g., not mentioned whether the electromyographic method was invasive or surface, or whether MRI or CT required anaesthesia), wrong population (*in silico* studies or a study population other than canids), wrong source of literature (not considered to be original quantitative research e.g., book chapters or reviews), intervention on cadavers, language other than English, wrong year of publication and study duplicate (i.e., the search both included a proceeding and an article of the same study). If full text versions of studies were not available online, and there was no response from direct contact to the authors after two attempts over 1 month, or if it was impossible to find contact information for authors *via* Google Scholar (www.scholar.google.com, RRID:SCR_008878), records were excluded as “not available.”

### 2.6. Data charting process

A data extraction table was jointly developed by all three reviewers to determine which variables to extract. Categories of muscle function assessment methods were continuously added to the table as they were identified from the eligible studies. Independently, two reviewers continuously charted data from each eligible study. At the end of data extraction, the data-charting forms were compared, and disagreements were resolved by consensus or, if consensus could not be reached, with input from the third reviewer. Authors were not contacted for clarification or to obtain additional information on incomplete studies.

### 2.7. Data items

Information collected from each study included author(s), publication title, year of publication, journal, one or more muscle function assessment methods, study population details, country, context (e.g., disease), aim/objective, intervention, and outcome/key findings. The final version of the data extraction form of all included studies is available online in Appendix II (https://osf.io/7j4yk).

### 2.8. Synthesis of results

The results were presented descriptively in tables and supplemented with appropriate graphical presentations. When the number of observations within each outcome measure was higher than 18, the most frequent results were presented. Studies were grouped by the type of muscle function assessment method(s) they included, allowing identification and mapping of the range of muscle function assessment methods currently applied in dogs. Further, the studies were grouped by the journals they were published in, by the countries and by the context of the studies (specific diseases or basic research). Information on the clinical relevance and clinical applicability of the identified methods was obtained by two clinical experts (JM and AV) who were asked to grade the level of clinical relevance of the methods: Grading 1–4 (poor, fair, good, excellent) for the potential quality of information provided to the clinician on muscle function in dogs with CCLD. Correspondingly, two experts in biomechanics (TA and MKZ) were asked to grade the level of applicability (method compliance) of the methods, including cost, training of staff, space requirements, time requirements etc. Method applicability was also graded from 1 to 4 (poor, fair, good, excellent). The clinical value gradings were then plotted against the method applicability gradings to identify methods with high value/high applicability, high clinical value/low applicability etc. During the method grading, the experts did not know the frequency of the identified methods applied in the included studies. The documents that the experts were given before grading can be seen online in Appendix III (https://osf.io/gf64p).

## 3. Results

### 3.1. Selection of sources of evidence

**Figure 1:**
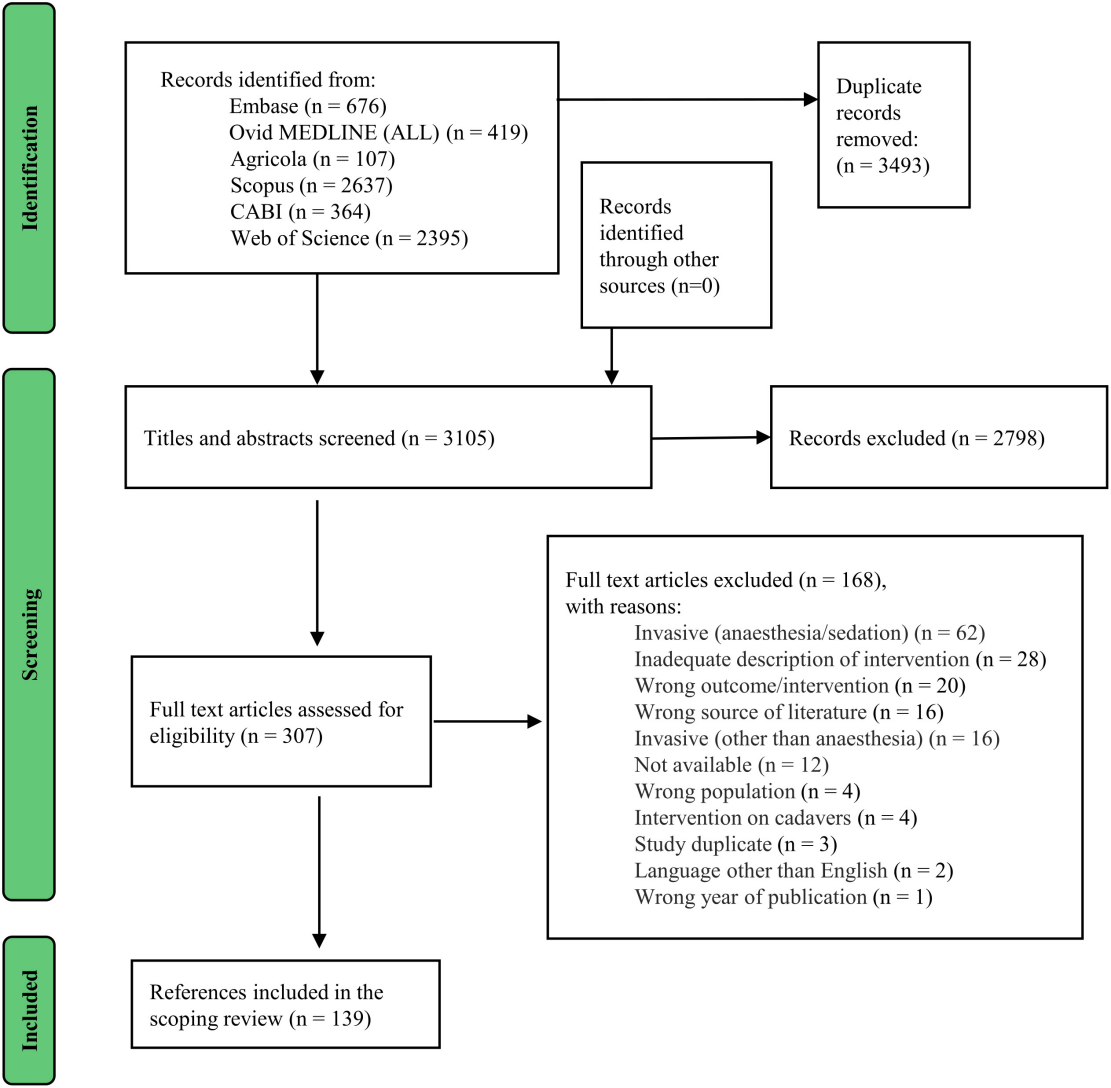
Flow diagram of the selection process (19, 21).

In total, 3,105 citations were identified from searches of the electronic databases and 139 studies were considered eligible for inclusion in this scoping review (Figure 1).

### 3.2. Characteristics of sources of evidence and synthesis of results

**Figure 2:**
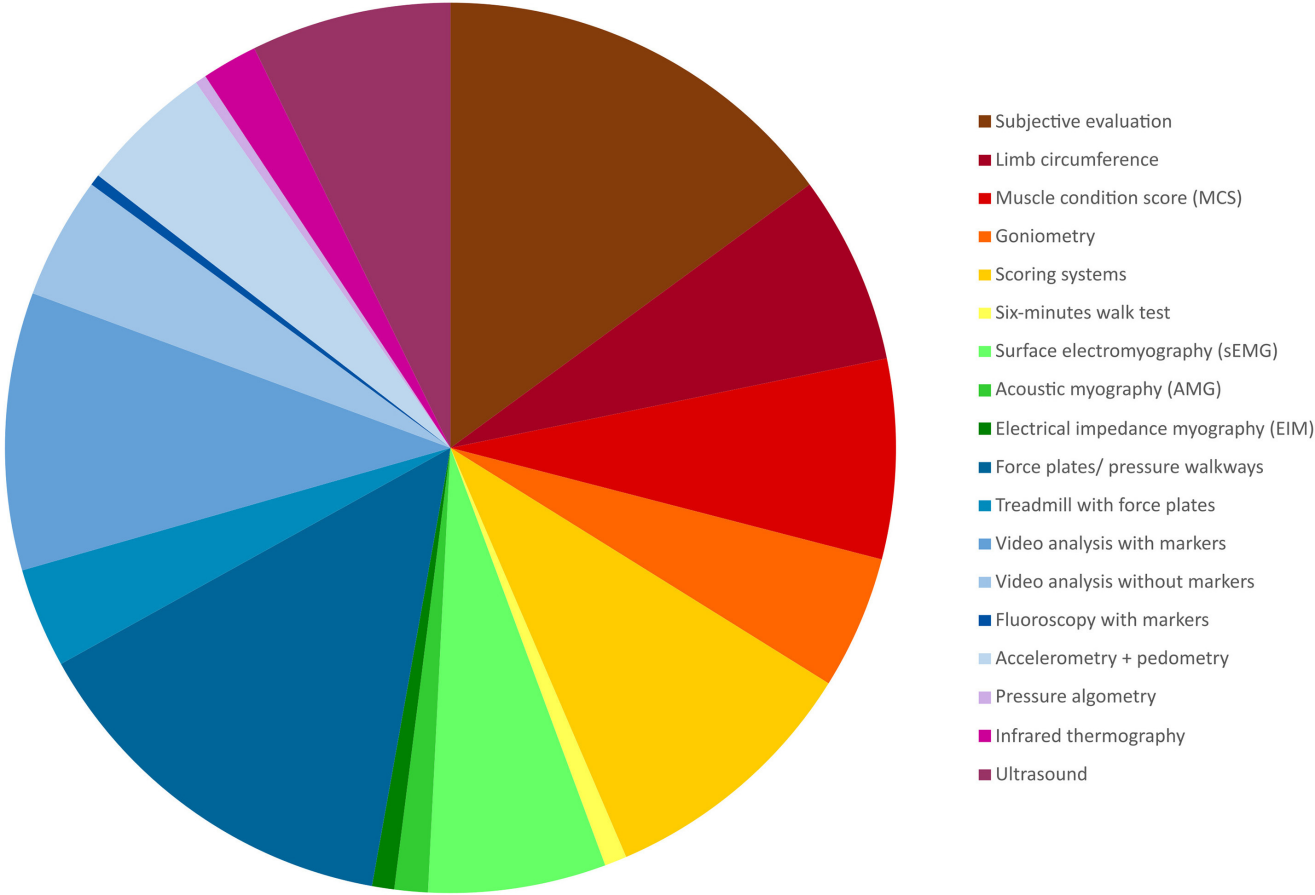
Pie chart visualising the distribution of the identified method categories based on all observations (*n* = 248).

The characteristics and relevant data charted from each of the included sources is provided in Appendix II, available online (https://osf.io/7j4yk). Eighteen different muscle function assessment categories were identified among the included studies and the percentage distribution of all observations (*n* = 248) is shown (Figure 2): subjective evaluation (consisting of a synthesis of multiple clinical assessments e.g., observation of gait or identification of muscle atrophy without any grading) (37/248, 14.9%), limb circumference (17/248, 6.9%), muscle condition score (MCS) (18/248, 7.3%), goniometry (12/248, 4.8%), scoring systems (lameness/pain scores etc.) (24/248, 9.7%), 6-min walk test (2/248, 0.8%), surface electromyography (sEMG) (16/248, 6.5%), AMG (3/248, 1.2%), electrical impedance myography (EIM) (2/248, 0.8%), force plate/force transducer/instrumented carpet/pressure walkway (35/248, 14.1%), treadmill with force plates (9/248, 3.6%), video analysis with markers (25/248, 10.1%), video analysis without markers (11/248, 4.4%), fluoroscopy with markers (1/248, 0.4%), accelerometry and pedometry (12/248, 4.8%), pressure algometry (1/248, 0.4%), infrared thermography (5/248, 2.0%), or ultrasound (18/248, 7.3%).

The method category called “scoring systems” comprised several validated and non-validated scoring systems primarily focusing on lameness or pain. This category (scoring systems), representing 24 of the included studies (three of them applying two scoring systems), was divided into 14 subcategories (Table 2).

**Table 2:** Unfolding the method category “scoring systems” including 24 studies (XLMTM: X-linked myotubular myopathy).

**Scoring system**	**Scale**	**References**
Canine acute pain scale	0–4	(22)
Helsinki chronic pain index	0–44	(23, 24)
Glasgow composite measure pain scale	0–24	(25)
Liverpool osteoarthritis in dogs (LOAD)	0–52	(26)
The finnish canine stifle index	0–263	(27)
Canine brief pain inventory/visual analogue scale (VAS)	0–100	(24, 28, 29)
Canine orthopaedic index scores	0–100%	(30)
Scoring sheet for the canine dystrophic phenotype	0–22	(31)
Neuromuscular assessment score for XLMTM	1–10	(32)
Disability index (DI) score	1–9	(33)
Lameness	0–4	(6, 34–38)
Lameness or pain	0–5	(39–41)
Lameness, pain, weight bearing or disability	1–5	(40, 42, 43)
Motor deficits or movements	0–3	(35, 44)

The number of studies represented within each muscle function assessment category varied from one to 37, and the number of muscle function assessment methods per study varied from one to five. The mean (SD) size of the study population in the included studies was 35.1 (76.3), the lower quartile was 6, the median 11.5, and the upper quartile of the study population was 25.75.

The most frequent context area among the included studies was basic research on healthy dog(s) (48 studies) followed by CCLD (16 studies) (Table 3).

**Table 3:** The context areas in the included studies.

**Context (e.g., disease)**	**Studies, *n***
Basic research, healthy dog(s)	48
Cranial cruciate ligament disease (CCLD)	16
Forelimb disorders (elbow, shoulder etc.)	10
Muscular dystrophy (Golden retriever or Duchenne)	19
Hindlimb disorders (not including CCLD)	10
Hip disorders	9
Myopathies	9
Spine/spinal cord disorders	6
Nutrient-related diseases	5
X-linked myotubular myopathy	5
Diseases represented by fewer than 3 articles (*n* = 7)	11

**Figure 3:**
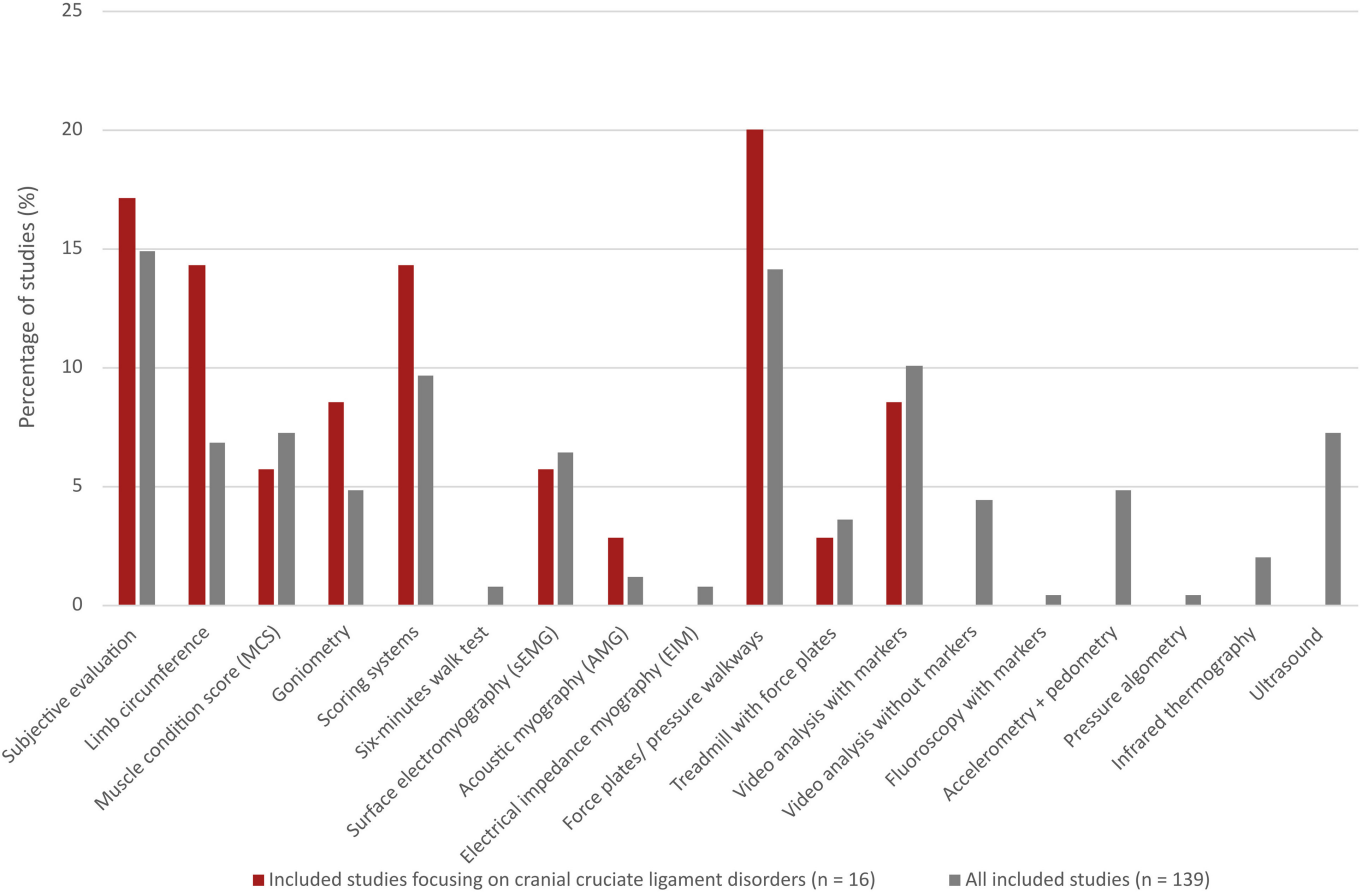
Percentage distribution of 18 different muscle function assessment methods among all the included studies (*n* = 139, grey bars) compared with the distribution in only a small part of these studies focusing on cranial cruciate ligament disease (*n* = 16, red bars).

The percentage distribution of the identified 18 muscle function assessment methods among all the included studies (*n* = 139) and studies focusing on CCLD [*n* = 16 (1, 6, 14, 34, 35, 39, 40, 45–53)] are shown in Figure 3. Ten out of the 18 muscle function assessment methods were applied in studies on CCLD. A greater representation of limb circumference, goniometry, scoring systems and force plates/pressure walkways was observed for CCLD studies when compared with all the included studies. Video analysis without markers, accelerometry/pedometry, and ultrasound were some of the methods that were not applied among the CCLD studies. Scoring systems were applied in five of the CCLD studies and covered simple lameness scores but with different scales (6, 34, 35, 39, 40).

**Figure 4:**
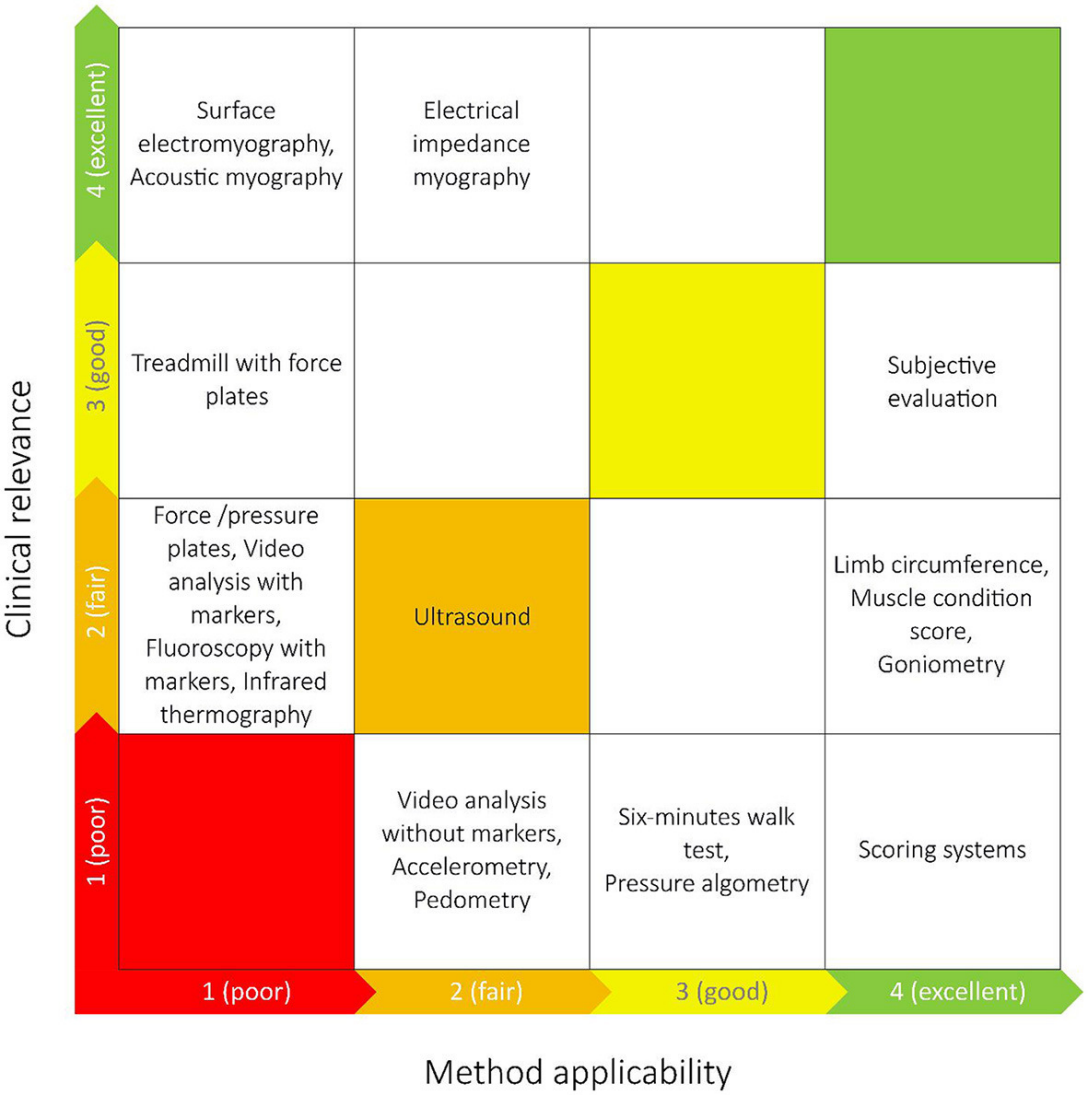
The grading of the identified muscle function assessment methods made by experts in biomechanics (*x*-axis) and clinical experts (*y*-axis) in relation to dogs with cranial cruciate ligament disease. The most optimal techniques are placed closer to the upper right corner, and the least optimal closest to the lower left corner.

The method grading made by clinical experts and experts in biomechanics are shown in Figure 4. Subjective evaluation, scoring systems, limb circumference, MCS and goniometry were graded as methods with high applicability. AMG, sEMG and EIM were graded as methods with the most clinically relevant outcome for dogs with CCLD.

**Figure 5:**
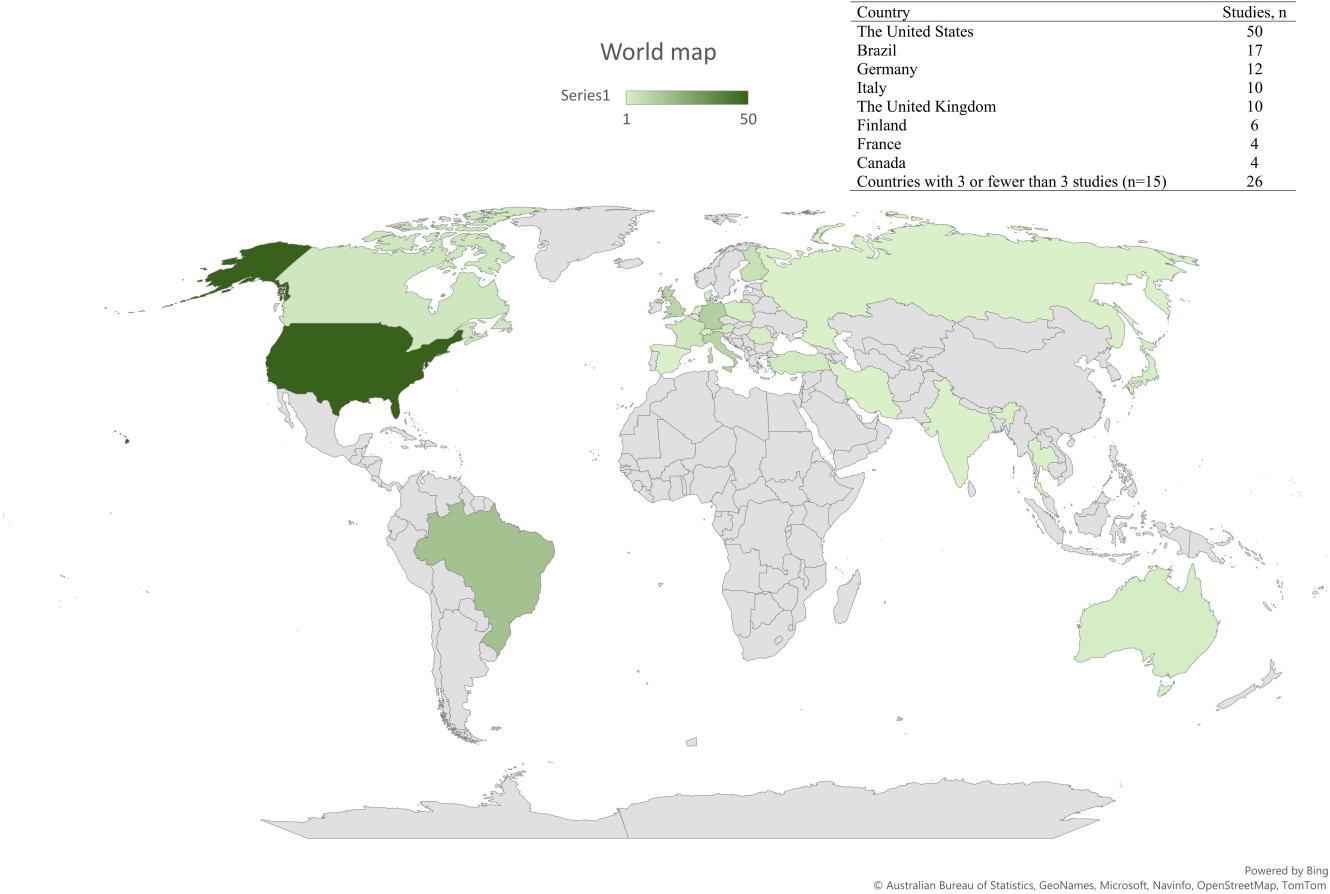
Geographical distribution of where the 139 included studies were conducted.

Figure 5 shows the geographical distribution of the included studies; 23 countries were represented. The most frequent countries were the United States (50 studies), Brazil (17 studies), Germany (12 studies), Italy (10 studies) and The United Kingdom (10 studies).

In general, the distribution of the methods applied in studies within the same country were as diverse as between countries. As an example, 17 studies included from Brazil covered 12 of the identified 18 method categories without any obvious links between the studies. However, there were exceptions for some of the countries: two out of three of the included studies from Austria (54, 55) were from the same group applying the same methods, and the two studies from Denmark used AMG (56, 57). Further, five out of six studies from Finland were from the same research group but applied different methods in the studies (6, 27, 35, 47, 58). Accordingly, the included 12 studies from Germany were conducted by four research groups (59–70). The German studies had a relatively high fraction of the objective, quantitative methods focusing on kinematics and kinetics e.g., six out of the nine studies applying a treadmill with force plates were German.

The 16 included studies focusing on CCLD were conducted in 9 different countries from five continents, and with the United States as the most represented country (*n* = 6).

There were 65 scientific journals represented among the included studies; the most frequent journals were PLOS ONE (12 studies), Veterinary Surgery (9 studies), Veterinary and Comparative Orthopaedics and Traumatology (8 studies), and Acta Veterinaria Scandinavica (8 studies) (Table 4).

**Table 4:** The most represented journals of the included studies.

**Journal**	**Studies, *n***
PLOS ONE	12
Veterinary surgery	9
Veterinary and comparative orthopaedics and traumatology	8
Acta veterinaria scandinavica	8
Animals	6
Frontiers in veterinary science	6
Journal of veterinary internal medicine	6
The veterinary journal	5
The Canadian journal of veterinary research	4
Comparative exercise physiology	4
American journal of veterinary research	4
Journals with 3 or fewer than 3 studies (*n* = 54)	67

## 4. Discussion

### 4.1. Summary of evidence

The aim was to identify and map current non-invasive muscle function assessment methods in dogs and to evaluate their applicability and relevance in clinical settings. We included 139 studies published in a variety of international peer-reviewed journals. Most of the identified studies represented basic research in healthy dogs. However, CCLD was the most frequently studied disease. The muscle function assessment methods in the included studies were divided into 18 categories and ranged from subjective examinations and simple measurements to complex and advanced methods. Muscle atrophy evaluation was one of the simple assessments. Muscle atrophy was identified by palpation without any grading (the method “subjective evaluation”), by muscle condition scoring the patient, and by measuring the limb circumference. Accordingly, lameness (i.e., altered muscle function in a limb) and pain were identified by clinical examinations and by scoring systems, the latter making the outcome quantitative. Pain scoring is not by definition considered directly related to muscle function. Nevertheless, if a dog perceives pain, this will often be coincident with reduced or altered muscle function. Further, pain assessment is defined as the fourth vital sign alongside temperature, pulse and respiration examinations, making pain assessment a way to capture more patients with altered muscle function (71). Unfolding the “scoring systems” category revealed that there was not a single standardised pain or lameness scoring system for dogs reported in the included studies. Further, there were no specific scoring systems for dogs with CCLD. As such, different scales of lameness grading were used (6, 34, 35, 39, 40), making it difficult to compare results between studies. Seven percent of the identified methods assessed muscle function by sEMG and AMG. Using these methods, the activity of specific muscles was investigated, and in some studies asymmetries between right- and left-sided muscles were quantified (46, 51). An integral part of most studies was gait analysis, since this term covers the entire range from visual observation of gait to quantified outcome measures as, for example, kinetic studies or kinematic studies using reflective markers attached to the dog (16). Subjective evaluation was the most frequently identified method category among all the included studies, while objective assessment using kinetic outcome measures was the second most frequent category in the included studies, and the most frequent category among studies focusing on CCLD.

The clinical relevance of evaluating surgical techniques and surgical outcomes for CCLD patients can explain why lameness, pain scoring, and biomechanical outcomes were represented among the included CCLD studies.

The clinical relevance of evaluating surgical techniques and surgical outcomes for CCLD patients may explain why lameness, pain scoring, and biomechanical outcomes were represented among the included CCLD studies, whereas the 6-min walk test and accelerometry/pedometry were not. This may be because the last-mentioned methods are primarily used to evaluate diseases that affect gait velocity, such as heart and respiratory diseases (72, 73). Dogs can establish compensatory movement patterns due to lameness, and thereby maintain their velocity. Thus, the 6-min walk test and accelerometry/pedometry are probably poor methods to evaluate surgical CCLD outcomes and therefore not represented among the included CCLD studies.

For most of the included studies, muscle function assessment was not the central purpose. Rather, the identified muscle function assessment methods were supplemental outcome measures. In general, the choice of muscle function assessment method in a given study depends on factors such as availability of equipment and the research question to be answered. It is possible that the percentage distribution of the identified methods in the present study would have been different if muscle function assessment were the main purpose of all the included studies.

The experts' grading of the muscle function assessment methods aimed at a clinical evaluation of the identified methods for dogs with CCLD. Given that CCLD was the most frequently represented disease state among the included studies, we found it appropriate to centre the expert evaluation on dogs with CCLD. The experts in biomechanics evaluated the subjective evaluation, scoring systems, limb circumference, MCS and goniometry to be the most applicable methods in clinical settings. The clinical experts evaluated sEMG, AMG, and EIM to be the methods with the most relevant information for dogs with CLLD. Subjective evaluation (consisting of a synthesis of multiple clinical assessments e.g., observation of gait or identification of muscle atrophy without any grading) was the muscle function assessment method placed closest to the top right corner in Figure 5 and thereby had the best combined grading made by the experts. The expert evaluation reflects the potential value of each method in the context of managing CCLD in practise, but the specific purpose of every study is key in the choice of muscle function assessment method.

The geographical distribution of the included studies was almost the same for the total included studies as for those focusing on CCLD. None of the included German studies focused on CCLD, but the same biomechanical method, e.g., the treadmill, were applied by other investigators on dogs with CCLD (49).

All the methods captured in this scoping review were indirect to varying degrees in the way they evaluated muscle function. However, the wide range of presented methods revealed good options to assess different aspects of muscle function in either research or clinical practises in the future. Given the high method applicability and clinical relevance of subjective evaluation, development of formalised, structured clinical assessment tools may be of value to future research.

### 4.2. Limitations

Our scoping review has some limitations. Firstly, despite using broad search terms in the search strategy, it is possible that eligible studies were missed by our search. Further, this scoping review did not specifically search for biomechanical studies as the objective was to identify studies assessing the muscles *per se*, and the search strategy was designed specifically to capture such studies. However, since many biomechanical studies were captured by our search, a wider search strategy including biomechanical terms could potentially have found more eligible studies and increased the overall frequency of methods including force plates and motion capture/movement analysis. Additionally, several studies were excluded due to non-invasiveness as an inclusion criterion. Our intention was to exclude invasive experimental procedures not transferable to clinical settings. Since sedation is commonly performed in clinical practise, it could be argued that it would have been acceptable and relevant to include studies with sedated or anaesthetized dogs, provided that the muscle assessment method itself was non-invasive. However, most of the studies which were excluded due to anaesthesia used CT or MRI, which is typically accessible only in larger clinics. The expert evaluations of the included muscle function assessment methods are subjective but reflect the potential value of each method in the context of managing CCLD in practice.

## 5. Conclusions

The width and depth of the literature on muscle function assessment methods in dogs was identified by this scoping review. The literature included simple case studies conducted in clinical settings as well as highly advanced basic research on experimental dogs. We observed that simple subjective evaluation was the most frequently used method for muscle function in dogs, and it was also the method with the best combined rating made by the experts in this scoping review. Kinetic methods were the most frequently reported in studies on dogs with CCLD. In total, 18 muscle function assessment categories were identified, with limited standardisation of muscle function assessment. This highlights the need for more and ideally high-quality research to establish consensus within muscle function assessment methods both in general and for specific patient groups, such as dogs with CCLD.

## Author contributions

KD was the primary person developing the review protocol and the search strategy, conducted the literature search, and the first draft of the manuscript. KD, TA, and MZ were involved in the eligibility screening, discussed the findings, extracted the relevant data, and discussed ideas on how to present data in the best way. JM and AV acted as expert clinicians in grading the muscle assessment methods. TA and MZ acted as experts in biomechanics. All authors discussed the results and contributed to the revision of the manuscript, read, and approved the submitted version.

## Supplementary material

Appendix I Search strategy (https://osf.io/sdnxj).

Appendix II Data extraction form (https://osf.io/7j4yk).

Appendix III Documents for the clinical experts and experts in biomechanics (https://osf.io/gf64p).
