# Corrigendum: Non-invasive methods to assess muscle function in dogs: a scoping review

**DOI:** 10.3389/fvets.2024.1365518

**Published:** 2024-02-06

**Authors:** Kathrine Højte Dahl, Mette Kreutzfeldt Zebis, Anne Désiré Vitger, James Edward Miles, Tine Alkjær

**Affiliations:** ^1^Department of Veterinary Clinical Sciences, University of Copenhagen, Copenhagen, Denmark; ^2^Department of Midwifery, Physiotherapy, Occupational Therapy and Psychomotor Therapy, University College Copenhagen, Copenhagen, Denmark; ^3^Institute of Sports Medicine Copenhagen, Copenhagen University Hospital–Bispebjerg and Frederiksberg, Copenhagen, Denmark; ^4^Department of Biomedical Sciences, University of Copenhagen, Copenhagen, Denmark; ^5^The Parker Institute, Bispebjerg-Frederiksberg Hospital, Copenhagen, Denmark

**Keywords:** dogs, muscle function, methods, non-invasive, cranial cruciate ligament rupture

In the published article, there was an error in Figure 4 as published. “Electrical impedance myography” was repeated by mistake the square furthest to the right in the second row from the top. This box should have instead read “Subjective evaluation”. The corrected Figure 4 and its caption appear below.

**Figure 4:**
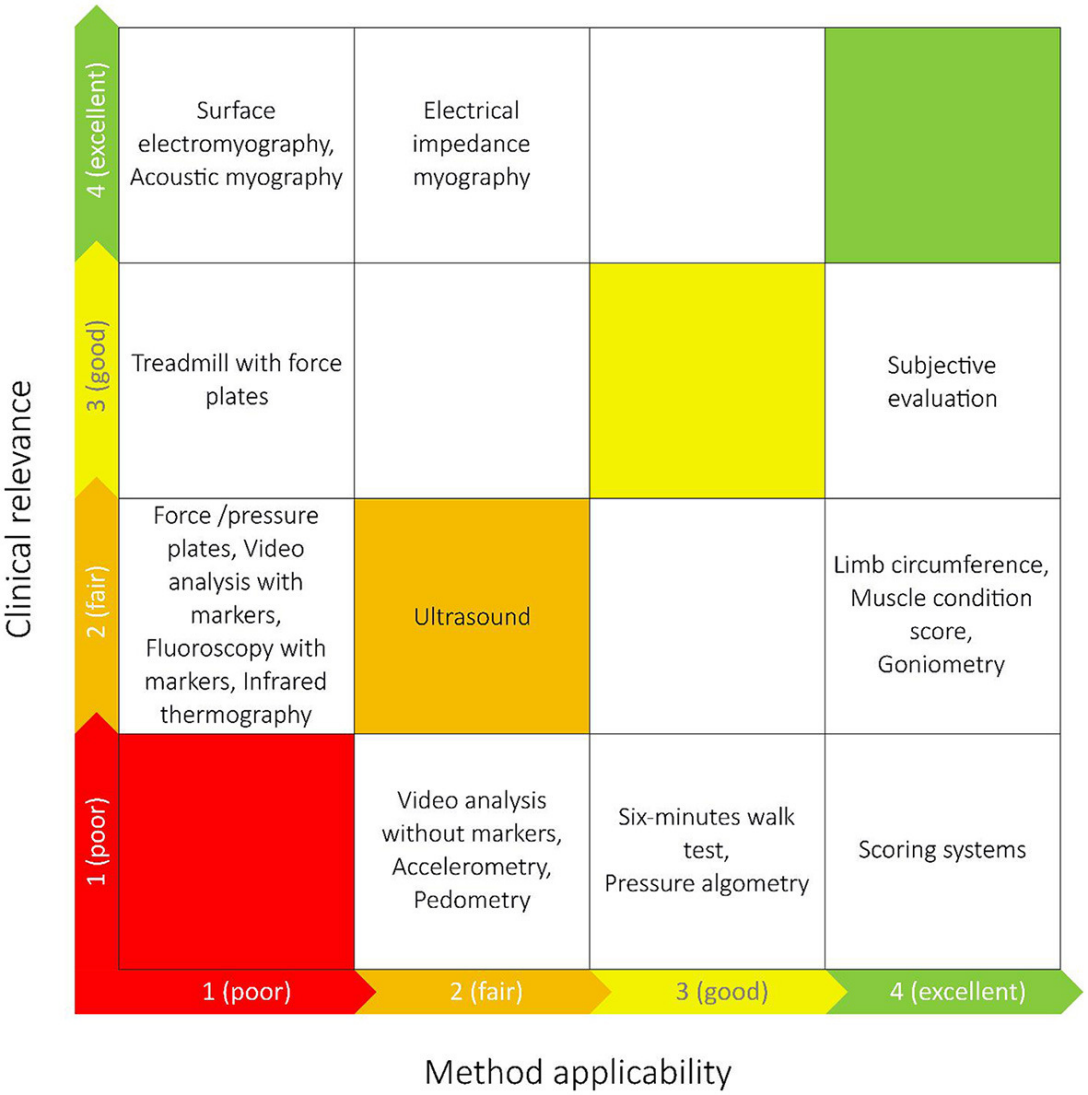
The grading of the identified muscle function assessment methods made by experts in biomechanics (*x*-axis) and clinical experts (*y*-axis) in relation to dogs with cranial cruciate ligament disease. The most optimal techniques are placed closer to the upper right corner, and the least optimal closest to the lower left corner.

The authors apologize for this error and state that this does not change the scientific conclusions of the article in any way. The original article has been updated.

