# Supplementary figures and images for: Non-invasive methods to assess muscle function in dogs: A scoping review
Source: Front Vet Sci. 2023 Jan 30;10:1116854. doi: 10.3389/fvets.2023.1116854 (PMC9923109; doi:10.3389/fvets.2023.1116854)

Appendix I

Embase:


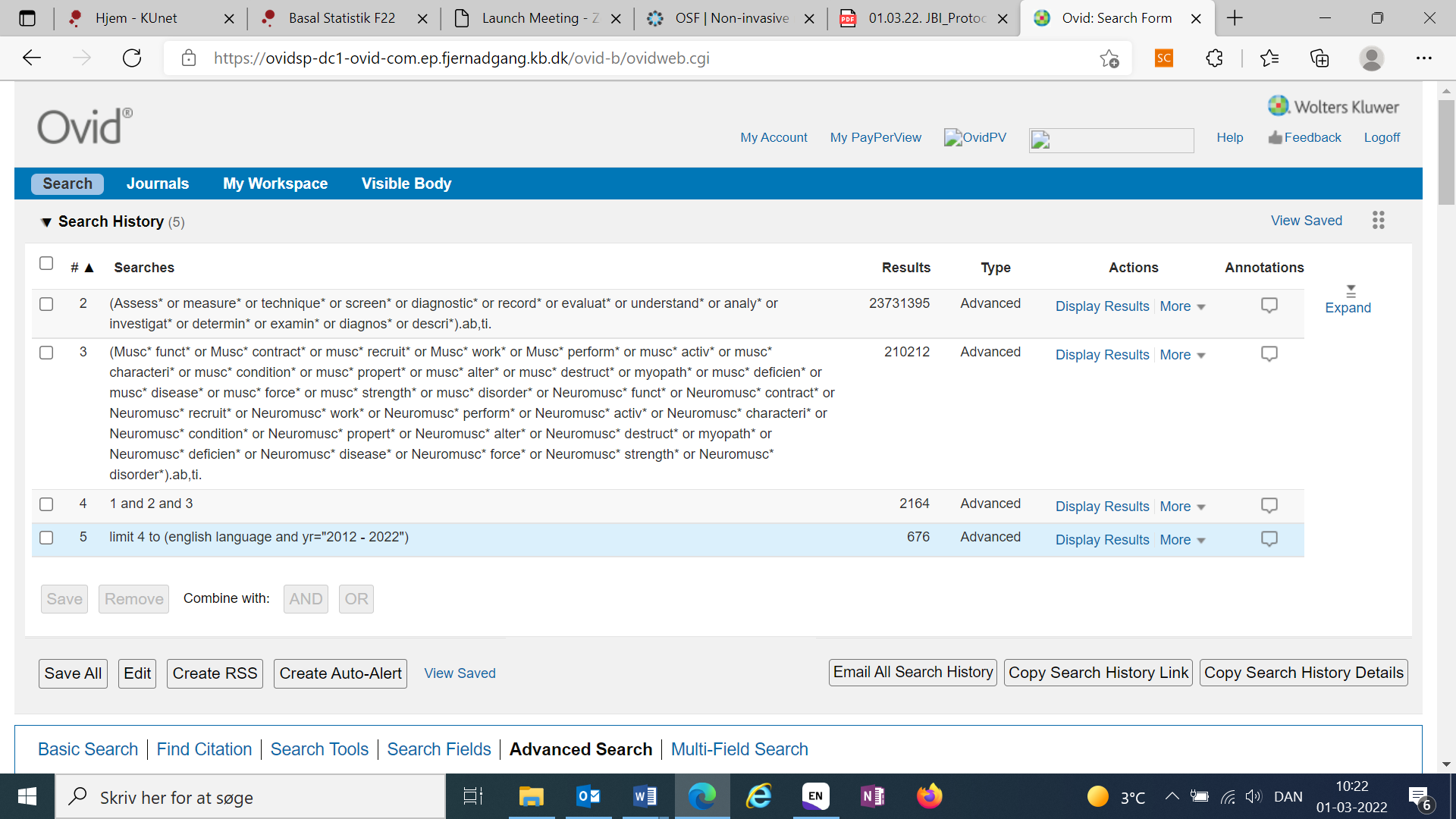


Web of science:


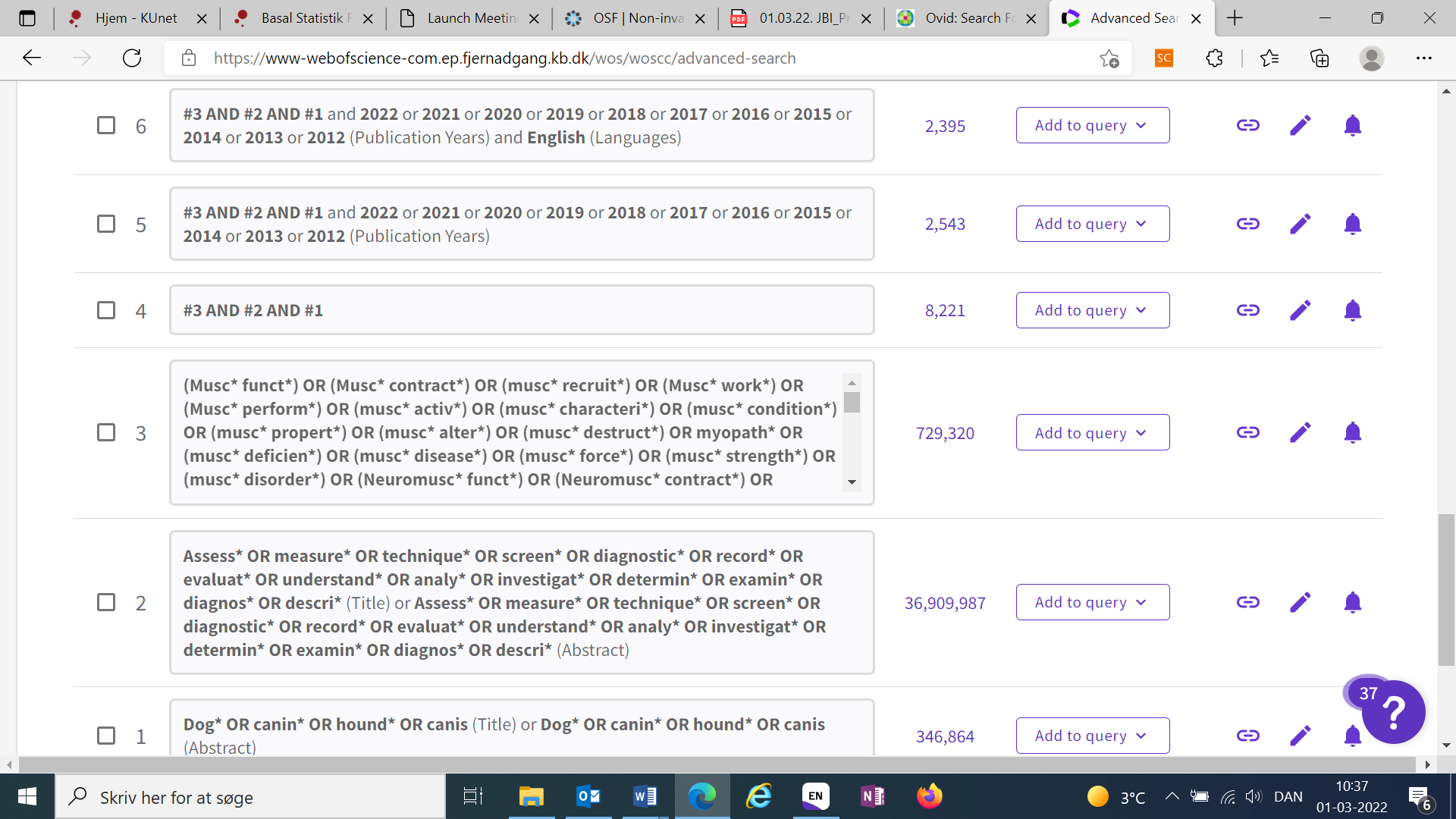

Supplement: Appendix I — Search strategy (https://osf.io/sdnxj). [file Data_Sheet_1.docx]
