# Supplementary material for: Non-invasive methods to assess muscle function in dogs: A scoping review
Source: Front Vet Sci. 2023 Jan 30;10:1116854. doi: 10.3389/fvets.2023.1116854 (PMC9923109; doi:10.3389/fvets.2023.1116854)
Supplement: Appendix II — Data extraction form (https://osf.io/7j4yk). [file Data_Sheet_2.docx]

**Catalogue for clinical experts**

The methods in this catalogue are the methods applied in the articles included in a coming scoping review about non-invasive methods to assess muscle function in dogs.

Please read the description about the different methods and afterwards grade each method from 1 to 4, where 1 is the lowest level of clinical relevance and 4 is the highest level of clinical relevance (the 4 levels are specified below). When grading the methods, it must be kept in mind that the grading is of the clinical relevance of the methods in relation to assessing muscle function in dogs with cranial cruciate ligament disorders (CCLD). Method compliance (i.e., how applicable the methods are, including cost, training of staff, space requirements, time requirements etc.) should **not** be considered in the grading.

Level 1 includes methods that only provide the clinician with poor information in the assessment of muscle function in dogs with CCLD.

Level 2 includes methods that provide the clinician with fair information in the assessment of muscle function in dogs with CCLD.

Level 3 includes methods that provide the clinician with good information in the assessment of muscle function in dogs with CCLD.

Level 4 includes methods that provide the clinician with excellent information in the assessment of muscle function in dogs with CCLD.

|  | Subjective evaluation (e.g. clinical examination) | Limb circumference | Muscle condition score | Scoring systems (Lameness / pain scores etc) | Pressure algometry | Infrared thermography | Six-minutes walk test | Ultrasound | Electrical impedance myography (EIM) | Surface electromyography (sEMG) | Acoustic myography (AMG) | Force plates/ force transducer / instrumented carpet/ pressure walkway | Treadmill with force plates | Goniometry | Accelerometry + pedometry | Video analysis no marksers | Markers + cameras | Markers + fluoroscopy |
| --- | --- | --- | --- | --- | --- | --- | --- | --- | --- | --- | --- | --- | --- | --- | --- | --- | --- | --- |
| **Level of clinical relevance** |  |  |  |  |  |  |  |  |  |  |  |  |  |  |  |  |  |  |
| Level 1 (poor) |  |  |  |  |  |  |  |  |  |  |  |  |  |  |  |  |  |  |
| Level 2 (fair) |  |  |  |  |  |  |  |  |  |  |  |  |  |  |  |  |  |  |
| Level 3 (good) |  |  |  |  |  |  |  |  |  |  |  |  |  |  |  |  |  |  |
| Level 4 (excellent) |  |  |  |  |  |  |  |  |  |  |  |  |  |  |  |  |  |  |

1. **Subjective evaluation**

Clinical examinations with palpation of the muscles, observation of the gait etc. is a subjective evaluation of a dog’s muscle function.


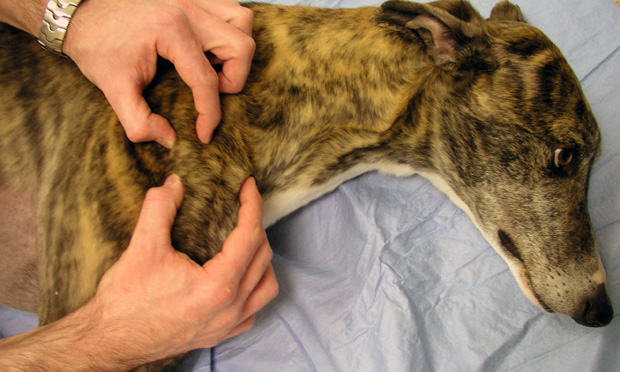


1. **Limb circumference**

Limb circumference (often thigh girth) is measured by soft measuring tape.


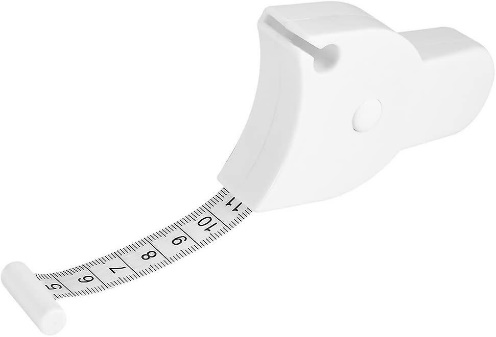


1. **Muscle condition score (MCS)**

Muscle condition score is a subjective scoring into 4 categories.


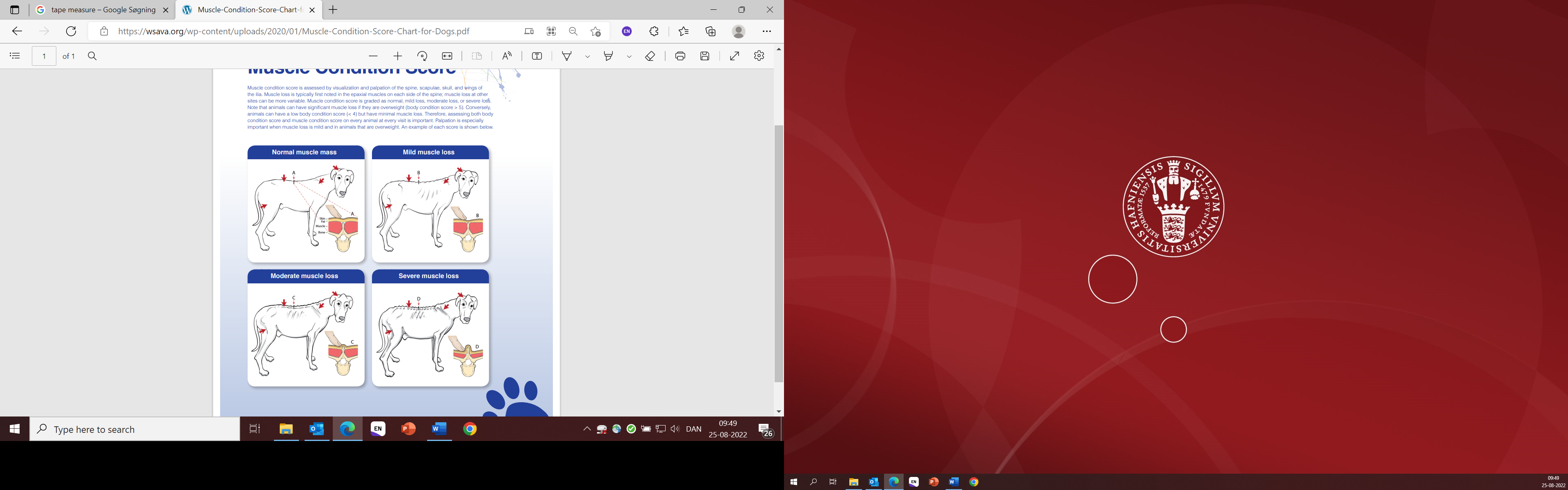


1. **Scoring systems (Lameness / pain scores etc)**

The scoring systems assess mostly dogs’ pain or lameness, but also weight bearing and motor deficits. This “method” includes all scoring systems found by the screening i.e., simple homemade grading scales and some validated questionnaires. For example, lameness scores, Visual Analogue Scale (VAS), Glasgow Composite Measure Pain Scale, Liverpool Osteoarthritis in Dogs (LOAD), and Canine Acute Pain Scale (two examples below).


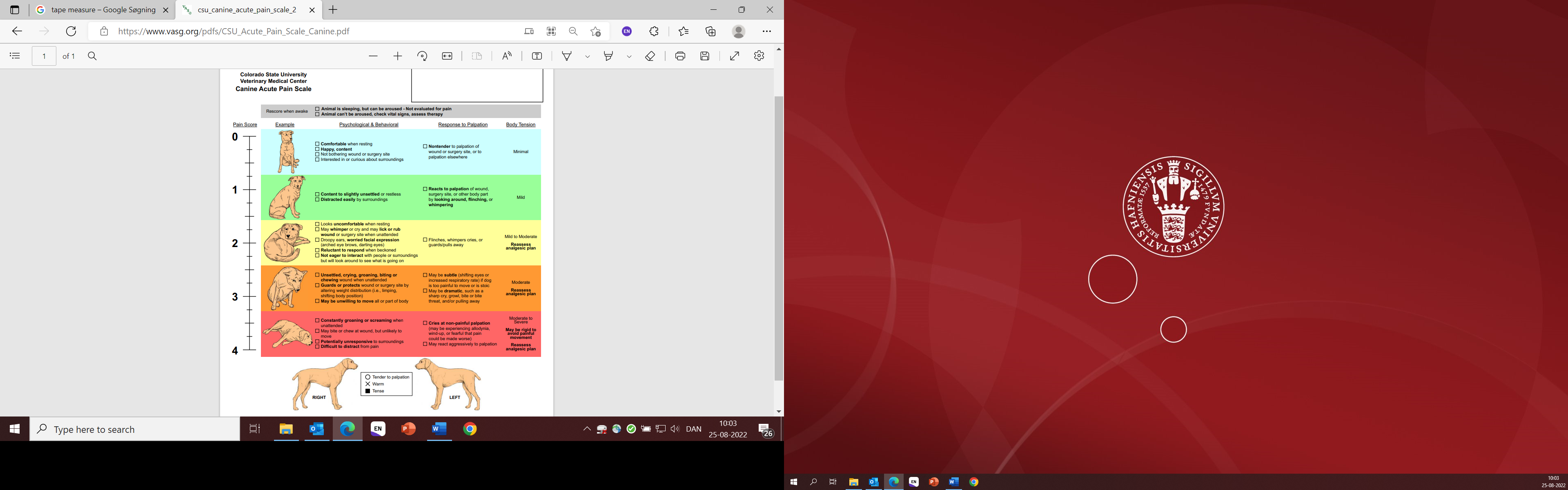


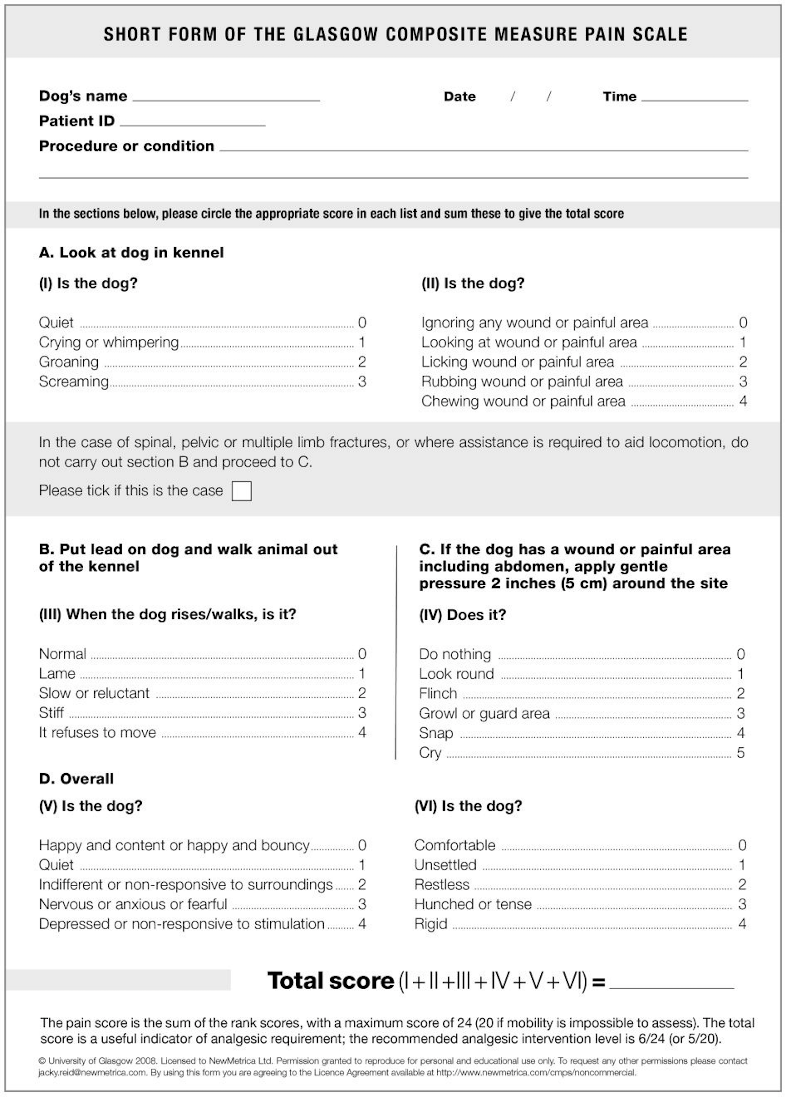


1. **Pressure algometry**

Pressure algometry is a method described to determine pressure pain threshold (PPT) in muscles by applying controlled pressure to a given body point.


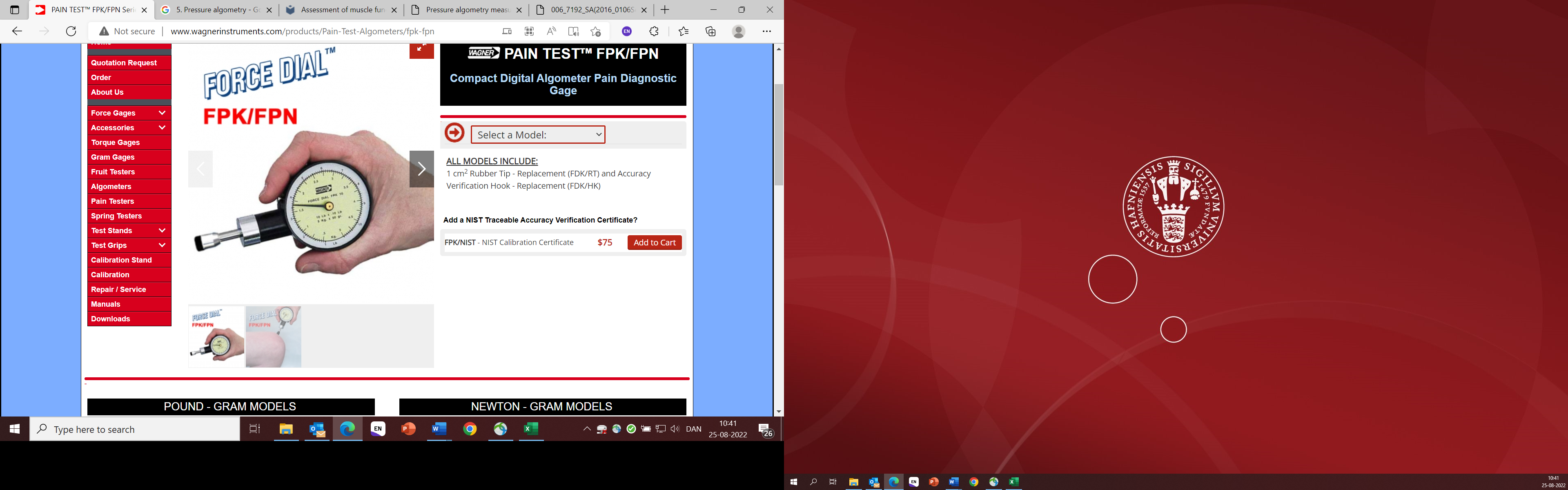


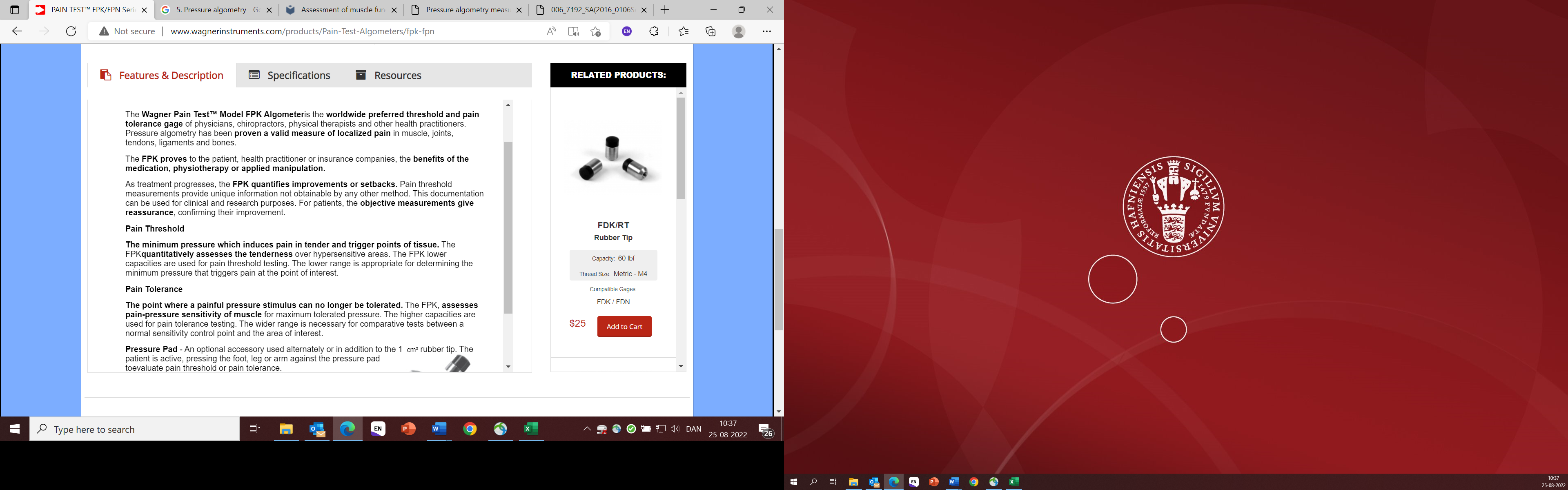


1. **Infrared thermography**

Infrared thermographic imaging (IRTG) is a technology used to monitor skin temperature and thereby exercising muscle power.


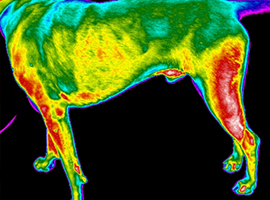


1. **Six-minutes walk test (6MWT)**

The 6-minute walk test (6MWT) is defined as the distance an individual can walk in 6 minutes.

1. **Ultrasound**

Ultrasound imaging uses sound waves to produce pictures. The interpreter then evaluates these pictures; do the muscles look healthy, damaged etc. Based on the pictures, measurement of e.g., muscle thickness can be made.


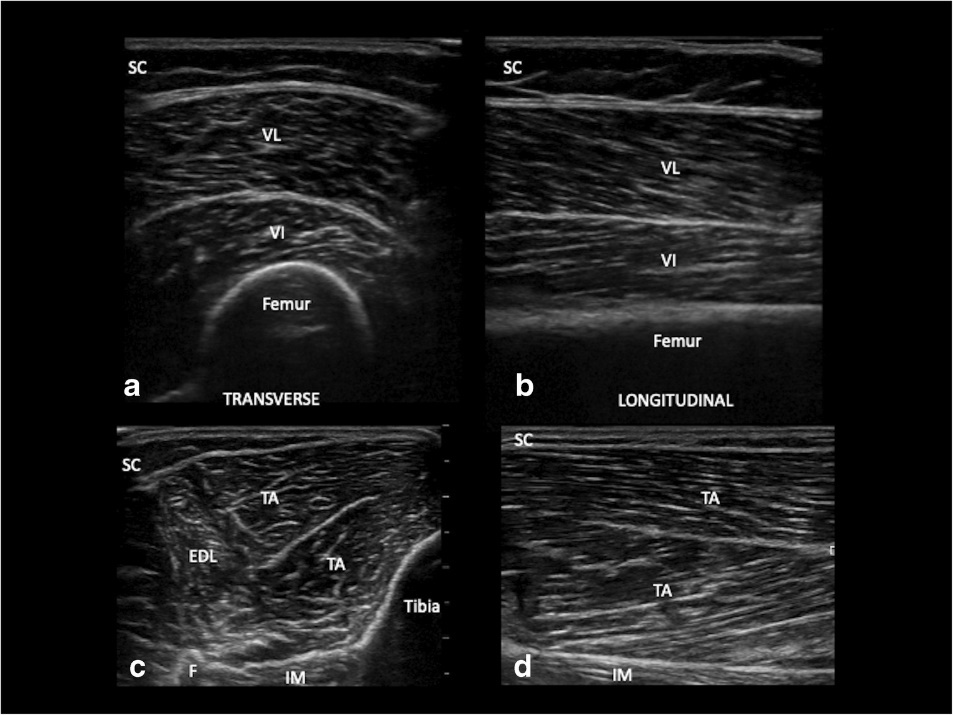


1. **Electrical impedance myography (EIM)**

Electrical impedance myography is a technique for the assessment of muscle health that is based on the measurement of the [electrical impedance](https://en.wikipedia.org/wiki/Electrical_impedance) characteristics of individual muscles or groups of muscles.

**
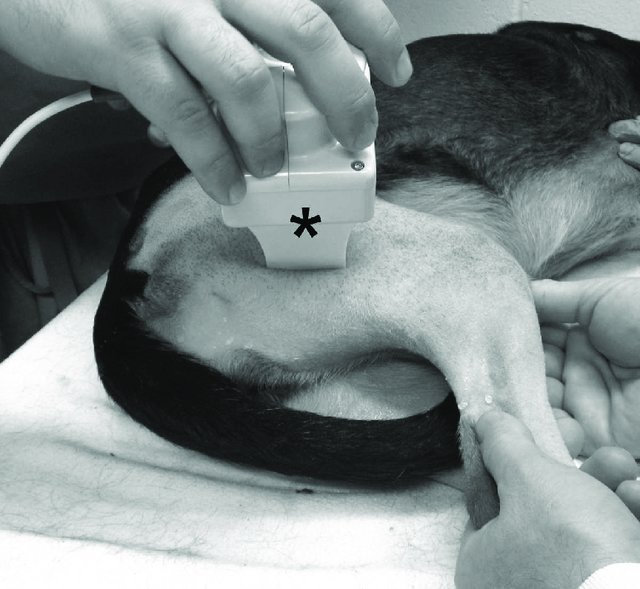
**

1. **Surface electromyography (sEMG)**

Surface electromyography records the electrical activity generated by muscle cells when these cells are activated. This is thereby an assessment of muscle activation by putting sensors on the skin above the muscles of interest.


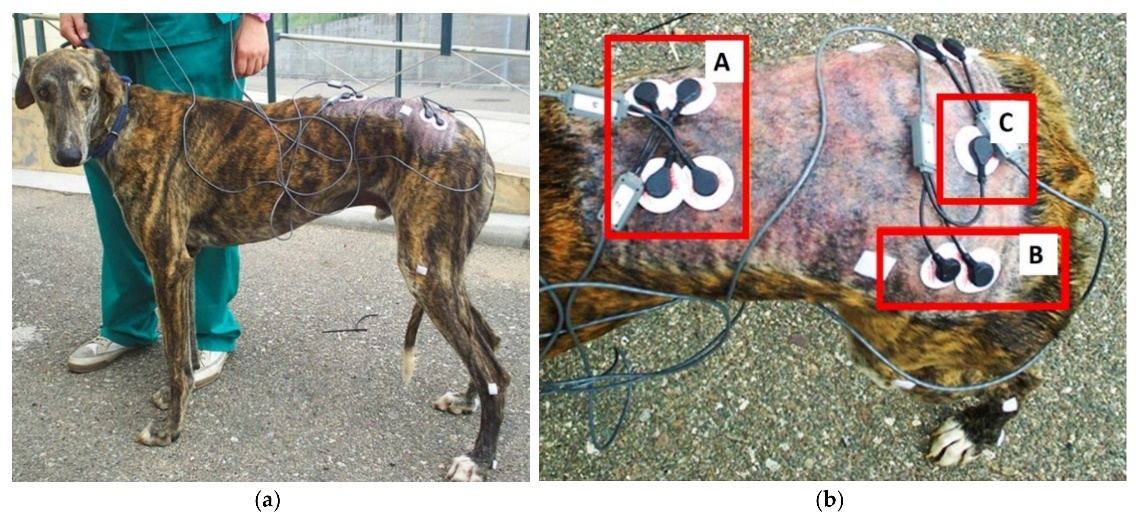


1. **Acoustic myography (AMG)**

Acoustic myography is a technique that measures the activity of muscle contraction by recording the low frequency sounds created during muscular activity. This is done by putting sensors on the skin above the superficial muscles of interest. This gives measurements on how the muscles work: the frequency in Hz and the amount of muscle fibres recruited.


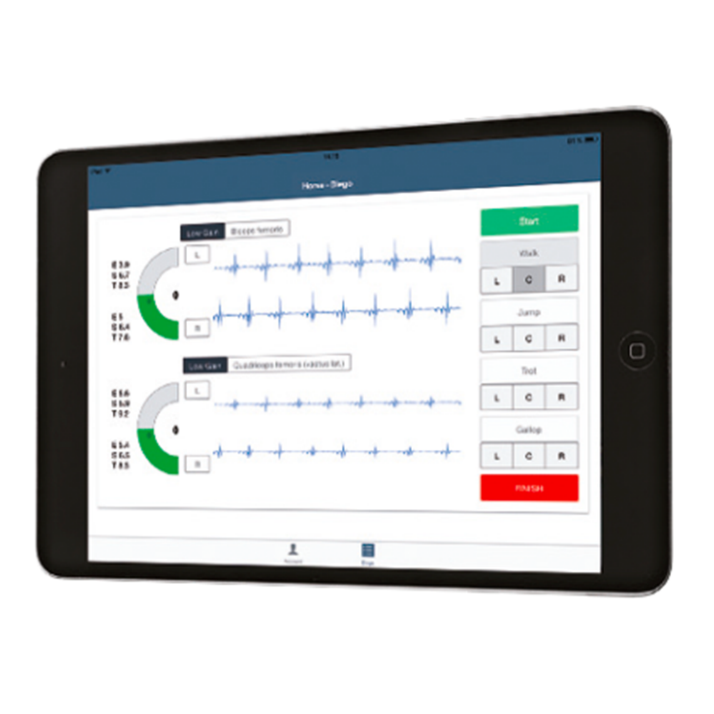


1. **Force plates / force transducer / instrumented carpet / pressure walkway**

Force plates / pressure walkways placed on the floor capture force and pressure, plus temporal (time) and spatial (distance) gait parameters in the evaluation of a dog’s gait.


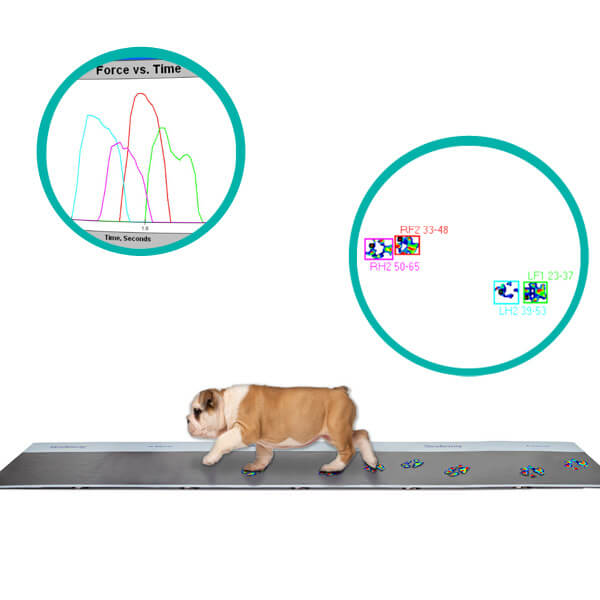


1. **Treadmill with force plates**

A treadmill with force plates sets the speed of the dog’s movements as part of the gait analysis. The treadmill is equipped with a calibrated pressure sensor matrix and mostly one or several synchronised cameras.


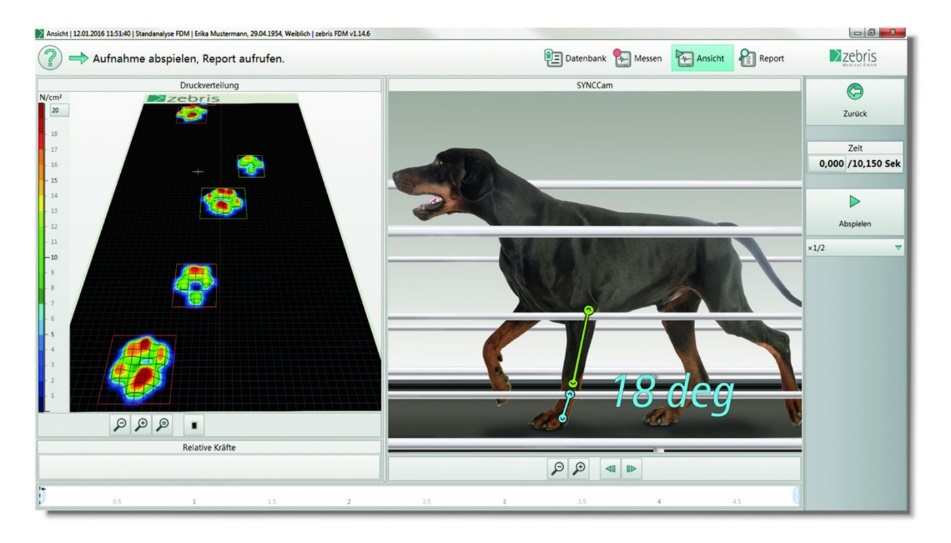


1. **Goniometry**

A goniometer measures the joint angle or range of motion of a joint.


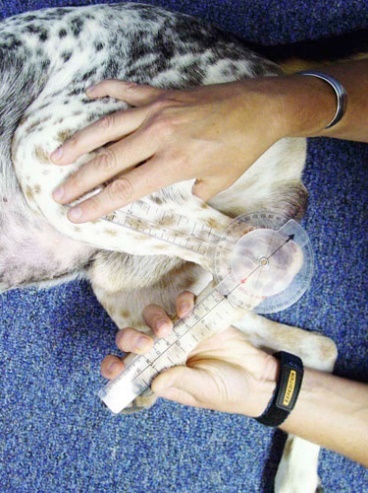


1. **Accelerometry + pedometry**

Accelerometers are movement monitors that can capture intensity of physical activity and thereby the change in velocity per unit time (m/s2). The acceleration is recorded in three axes.

Pedometry provides an estimate of the distance walked.


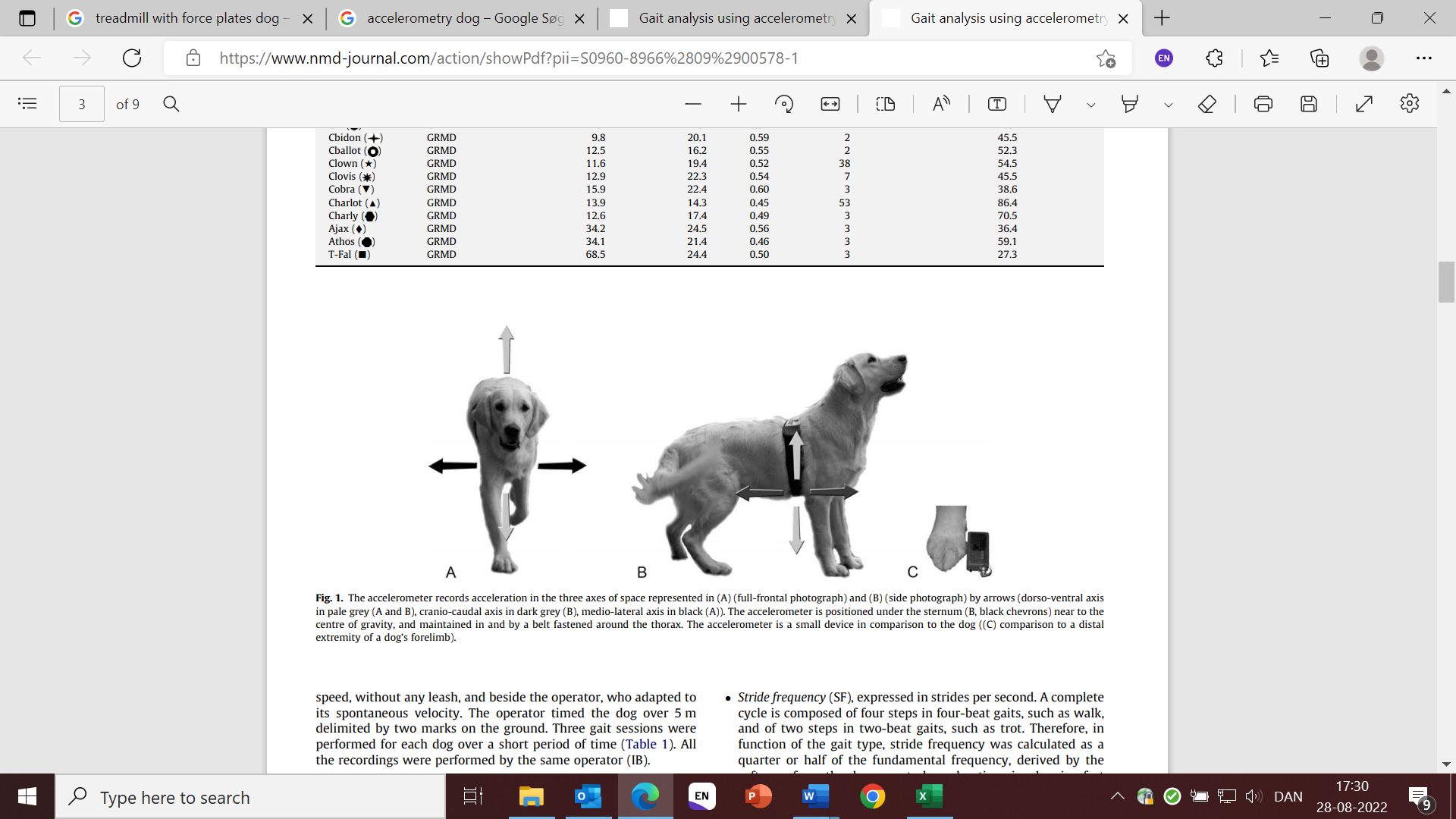


1. **Video analysis without markers**

Video analysis without the use of markers is a way to make a gait analysis by evaluating videos of a dog’s gait.


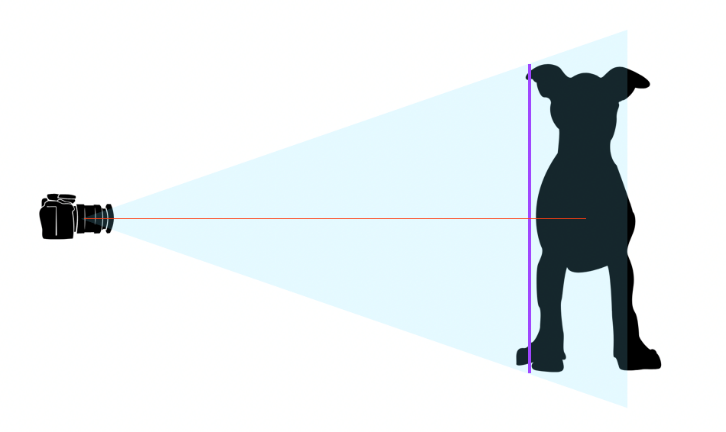


1. **Markers and camera**

This kind of gait analysis includes markers attached to the skin of the dog and cameras placed in a room to capture the movements of a dog and evaluate the gait in a quantitative way.


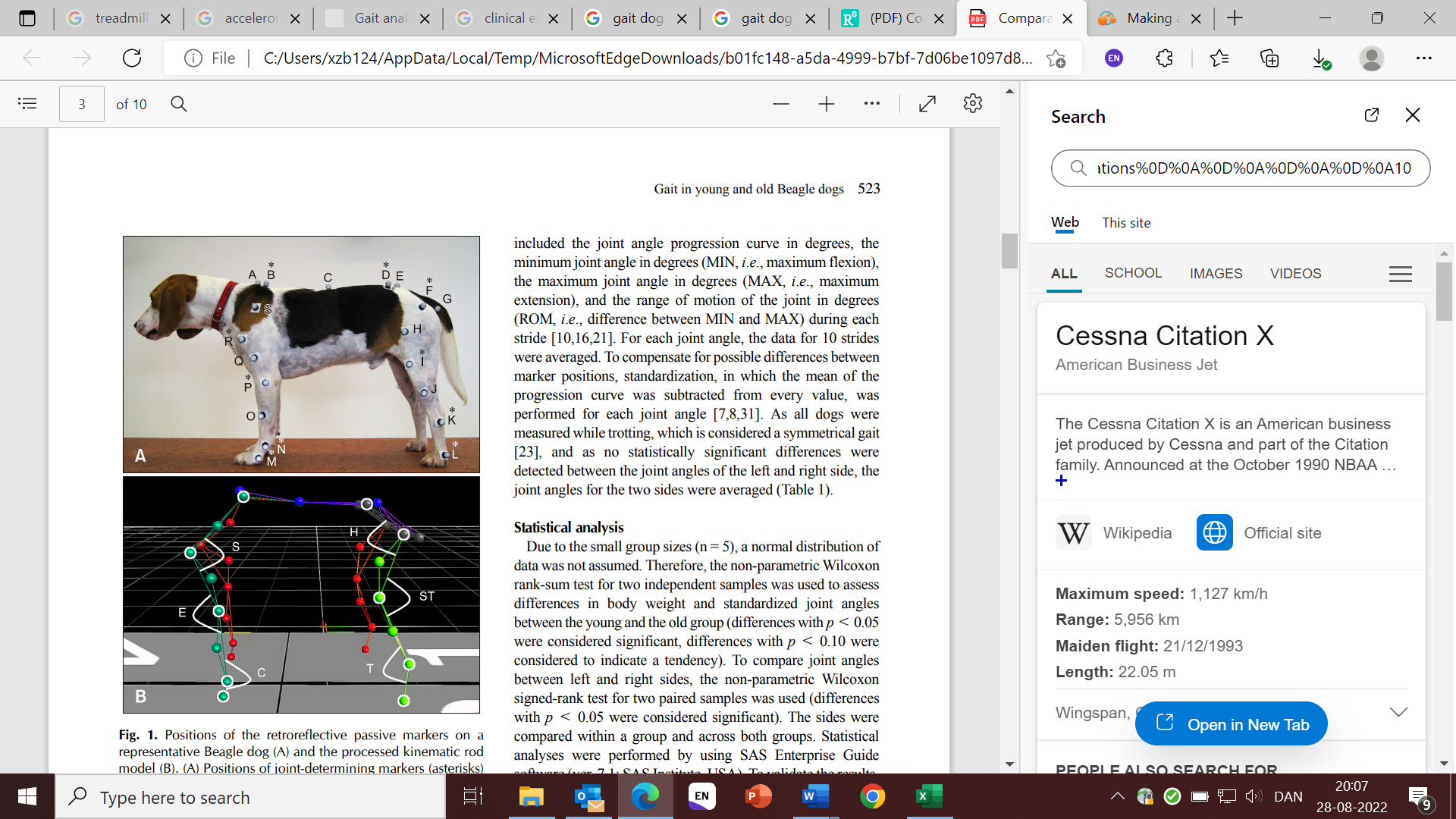


1. **Markers and fluoroscopy**

Fluoroscopy visualizes internal structures by using X-rays to produce a moving, real-time picture. This method makes it possible to make the placement of the markers precise and to use both the markers (quantitatively) and the visualization of the bones in the gait analysis.


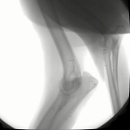


**References for the pictures (1 September 2022):**

1. <https://www.cliniciansbrief.com/article/orthopedic-examination-forelimb-dog>

2. [https://www.fruugo.dk/150cm60inch-tape-measure-sewing-tape-measure-soft-tape- measure/p-87299120-182103416?language=en&ac=croud&gclid=EAIaIQobChMI6bPvi77h-QIVAoODBx1sggBjEAkYByABEgIevvD_BwE&gclsrc=aw.ds](https://www.fruugo.dk/150cm60inch-tape-measure-sewing-tape-measure-soft-tape-%20%20%20%20%20measure/p-87299120-182103416?language=en&ac=croud&gclid=EAIaIQobChMI6bPvi77h-QIVAoODBx1sggBjEAkYByABEgIevvD_BwE&gclsrc=aw.ds)

3. <https://wsava.org/wp-content/uploads/2020/01/Muscle-Condition-Score-Chart-for-Dogs.pdf>

4. <https://www.vasg.org/pdfs/CSU_Acute_Pain_Scale_Canine.pdf>

[https://vetfocus.royalcanin.com/en/scientific/pain-assessment-in-the-dog-the-glasgow-pain- scale](https://vetfocus.royalcanin.com/en/scientific/pain-assessment-in-the-dog-the-glasgow-pain-%20%20%20scale)

5. <http://www.wagnerinstruments.com/products/Pain-Test-Algometers/fpk-fpn>

6. <https://www.k9strong.org/canine-digital-thermal-imaging-digatherm/>

8. <https://link.springer.com/article/10.1007/s11926-020-00947-y/figures/1>

9. <https://www.researchgate.net/publication/315632925_Non-invasive_evaluation_of_muscle_disease_in_the_canine_model_of_Duchenne_muscular_dystrophy_by_electrical_impedance_myography/figures>

10. <https://www.mdpi.com/2076-2615/10/6/968>

11. https://curo-diagnostics.com/about-us/

12. <https://www.tekscan.com/products-solutions/animal-gait-analysis>

13. <https://www.zebris.de/en/veterinary/canidgait-gait-analysis-for-dogs-measuring-system-for-diagnostics-therapy-and-rehabilitation>

14. https://veteriankey.com/assessing-and-measuring-outcomes/

15. https://www.nmd-journal.com/action/showPdf?pii=S0960-8966%2809%2900578-1

16. http://caninefitnessinnovations.com/2019/09/15/making-a-gait-analysis-video/

17.https://www.researchgate.net/publication/315825766_Comparative_kinematic_gait_analysis_in_young_and_old_Beagle_dogs

18. http://www.fluokin.de/

**Grading the level of method compliance by**

**experts in biomechanics**

The methods in the table below are applied in the articles included in a coming scoping review about non-invasive methods to assess muscle function in dogs.

The level of method compliance must be graded for all 18 methods. Method compliance means how applicable the methods are in clinical settings and the following should be considered: Cost of the equipment, space requirements, time requirements and potential training of staff as a necessity.

**Level 1** includes methods with low method compliance i.e., very complex methods that are laboriously applied in clinical settings (e.g. a method that is expensive, with large space or special conditions/environment – and time – and training requirements)

**Level 2** includes methods with medium-low method compliance (e.g., a method that is expensive but less than level 1, time-consuming and requires training, but with lower space requirements than level 1)

**Level 3** includes methods with medium-high method compliance (e.g., a method that are somewhat cheaper and less skill-demanding than what those at level 2 require)

**Level 4** included methods with high method compliance i.e., simple methods that are easily applied in clinical settings (e.g., a method that is cheap, fast and with very simple space - and training requirements)

|  | Subjective evaluation (e.g. clinical examination) | Limb circumference | Muscle condition score | Scoring systems (Lameness / pain scores etc) | Pressure algometry | Infrared thermography | Six-minutes walk test | Ultrasound | Electrical impedance myography (EIM) | Surface electromyography (sEMG) | Acoustic myography (AMG) | Force plates/ force transducer / instrumented carpet/ pressure walkway | Treadmill with force plates | Goniometry | Accelerometry + pedometry | Video analysis no marksers | Markers + cameras | Markers + fluoroscopy |
| --- | --- | --- | --- | --- | --- | --- | --- | --- | --- | --- | --- | --- | --- | --- | --- | --- | --- | --- |
| Level of clinical relevance |  |  |  |  |  |  |  |  |  |  |  |  |  |  |  |  |  |  |
| Level 1 (poor) |  |  |  |  |  |  |  |  |  |  |  |  |  |  |  |  |  |  |
| Level 2 (fair) |  |  |  |  |  |  |  |  |  |  |  |  |  |  |  |  |  |  |
| Level 3 (good) |  |  |  |  |  |  |  |  |  |  |  |  |  |  |  |  |  |  |
| Level 4 (excellent) |  |  |  |  |  |  |  |  |  |  |  |  |  |  |  |  |  |  |
